# An Unexpected Case of Posterior Reversible Encephalopathy Syndrome

**DOI:** 10.7759/cureus.10129

**Published:** 2020-08-30

**Authors:** Hassan Baig, Mohammad H Ashraf, Mohammad Ahmad Ashraf, Mujahid A Khan, Mohamed Nauzan Mohamed Nazeer

**Affiliations:** 1 Department of General Surgery, Ninewells Hospital, Dundee, GBR; 2 Department of General Surgery, University of Dundee, Dundee, GBR; 3 Department of Neurology, University of Glasgow, Glasgow, GBR; 4 Department of General Surgery, University Hospital Hairmyres, Glasgow, GBR; 5 Department of Neurology, The James Cook University Hospital, Middlesbrough, GBR

**Keywords:** posterior reversible encephalopathy syndrome (pres), encephalopathy, visual disturbance, posterior circulation stroke, visual field loss, vascular dementia, cerebrovascular accident (stroke)

## Abstract

This study presents an 86-year-old gentleman who was admitted under the acute stroke team for a possible posterior cerebral infarct. Radiologic imaging revealed that the diagnosis was in fact posterior reversible encephalopathy syndrome (PRES). Through a process of elimination by means of investigations, the most likely cause was found to be mild hypertension on a background of vascular dementia causing a possible exacerbation of PRES symptoms. A multidisciplinary approach was found to be beneficial, providing safe and effective care for this patient, allowing a brief recovery period and restoration of baseline function and thus minimising permanent sequelae.

## Introduction

Posterior reversible encephalopathy syndrome (PRES) is a clinico-radiologic diagnosis typically characterised by headaches, seizures, visual disturbances and altered consciousness. Currently, there is no diagnostic criteria for this syndrome. There are several factors that can cause PRES, but it is most commonly triggered by acute hypertensive episodes [1,2]. Development of PRES has also been associated with acute and chronic kidney disease; however, there have been reports of PRES occurring due to autoimmune diseases, such as systemic lupus erythematosus in addition to exposure to various different immunosuppressive medications [3]. Thus, the aetiology of this condition remains relatively non-specific, and often difficult to isolate.

Hypertension (blood pressure exceeding 140 mmHg systolic and 90 mmHg diastolic) is estimated to affect more than 30% of the global population, contributing towards 12.8% of all worldwide deaths [4,5]. Whilst having elevated blood pressure does not routinely cause symptoms, chronically raised blood pressure is a major risk factor for a substantial number of conditions, such as coronary artery disease, hypertensive retinopathies and stroke.

Diagnosing PRES can be difficult due to the vague clinical presentation, which can be associated with a variety of different conditions. PRES is commonly mistaken for intracerebral haemorrhage and stroke; however, the diagnosis is confirmed using radiographic imaging. The pathophysiology is not entirely understood; however, it is thought that hypertensive episodes disrupt the blood-brain barrier due to endothelial injury and cytokine effects causing subcortical vasogenic oedema, subsequently detected on brain imaging [6]. Most notably, MRI scans reveal diffuse areas of white matter abnormalities, with bilateral involvement of the parietal and occipital lobes. Management for PRES primarily requires the treatment of the offending trigger; however, this can be challenging due to its uncertain aetiology.

## Case presentation

This study discusses the case of an 86-year-old man who was admitted to hospital for a three-week history of increased blurring of vision, vertigo on head movement and worsening cognition. On admission, his Montreal Cognitive Assessment score was 13/30 which was abnormal for him, as there was no previous history of significant cognitive impairment. Examination of the cranial nerves revealed horizontal nystagmus upon left lateral gaze as well as horizontal binocular diplopia, and incomplete abduction of his left eye. Initial visual field assessment showed a left inferior quadrantanopia. There were no motor or sensory deficit across all four limbs; however, the patient was noted to have left upper limb dysdiadochokinesia with past-pointing, pronator drift, and an ataxic gait. There was no reported headache or loss of consciousness.

The patient reported no history of recent illnesses. His past medical history included ischaemic heart disease, mild vascular dementia, hypertension and gastro-oesophageal reflux disease. The patient had no significant family history, minimal alcohol consumption and was an ex-smoker several decades ago. There was no history of foreign travel to indicate an infective cause for symptoms. His pre-admission medications were aspirin, amlodipine, atorvastatin and isosorbide mononitrate. 

Full blood count, and urea and electrolytes were found to be unremarkable. Liver function tests and serum ammonia were also within normal limits. Clinically, this patient was diagnosed with a posterior circulation stroke and as such, his antihypertensives were reduced to avoid hypoperfusion during his stroke disease.

**Figure 1:**
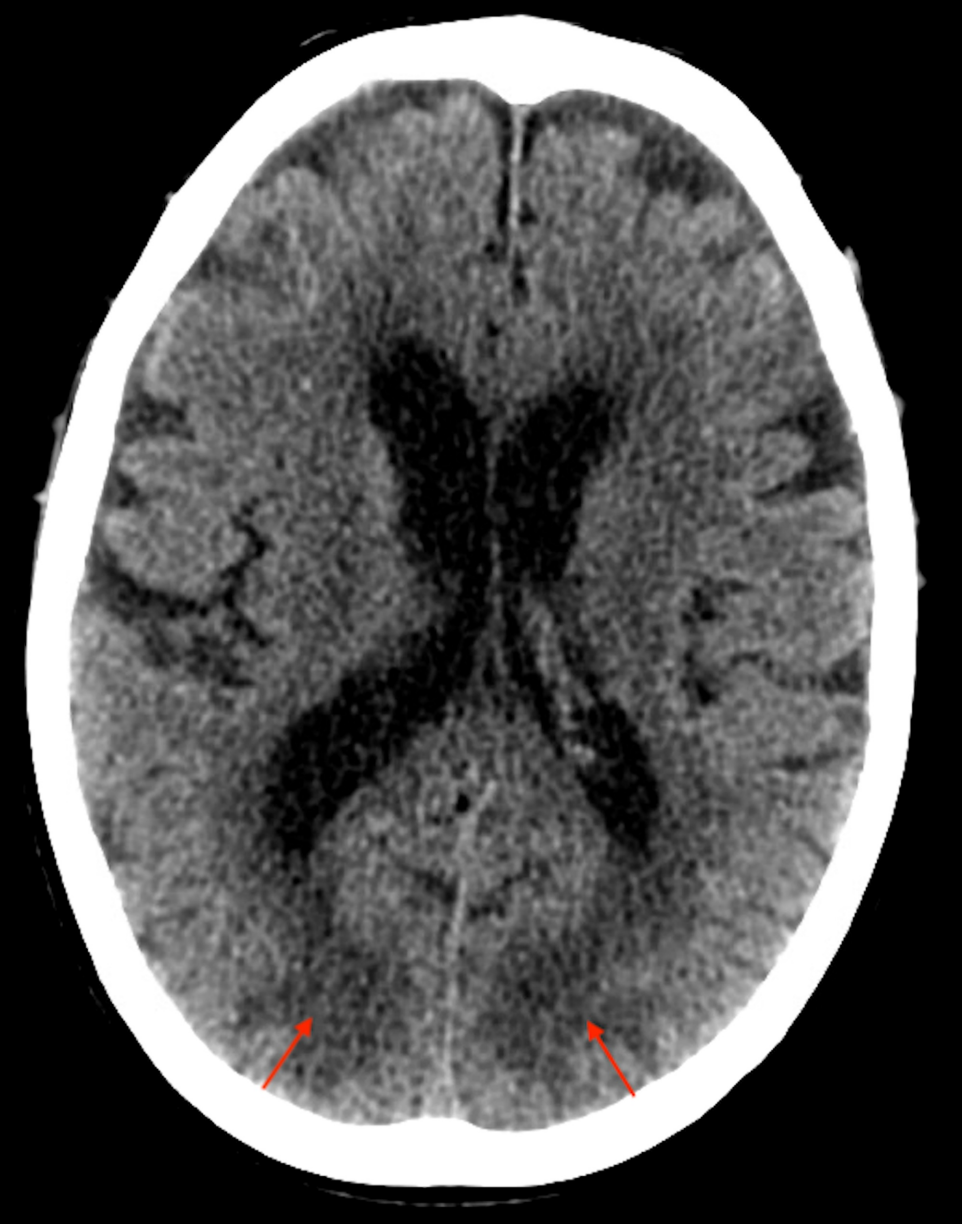
CT of the head reveals low attenuation changes in occipital lobes

**Figure 2:**
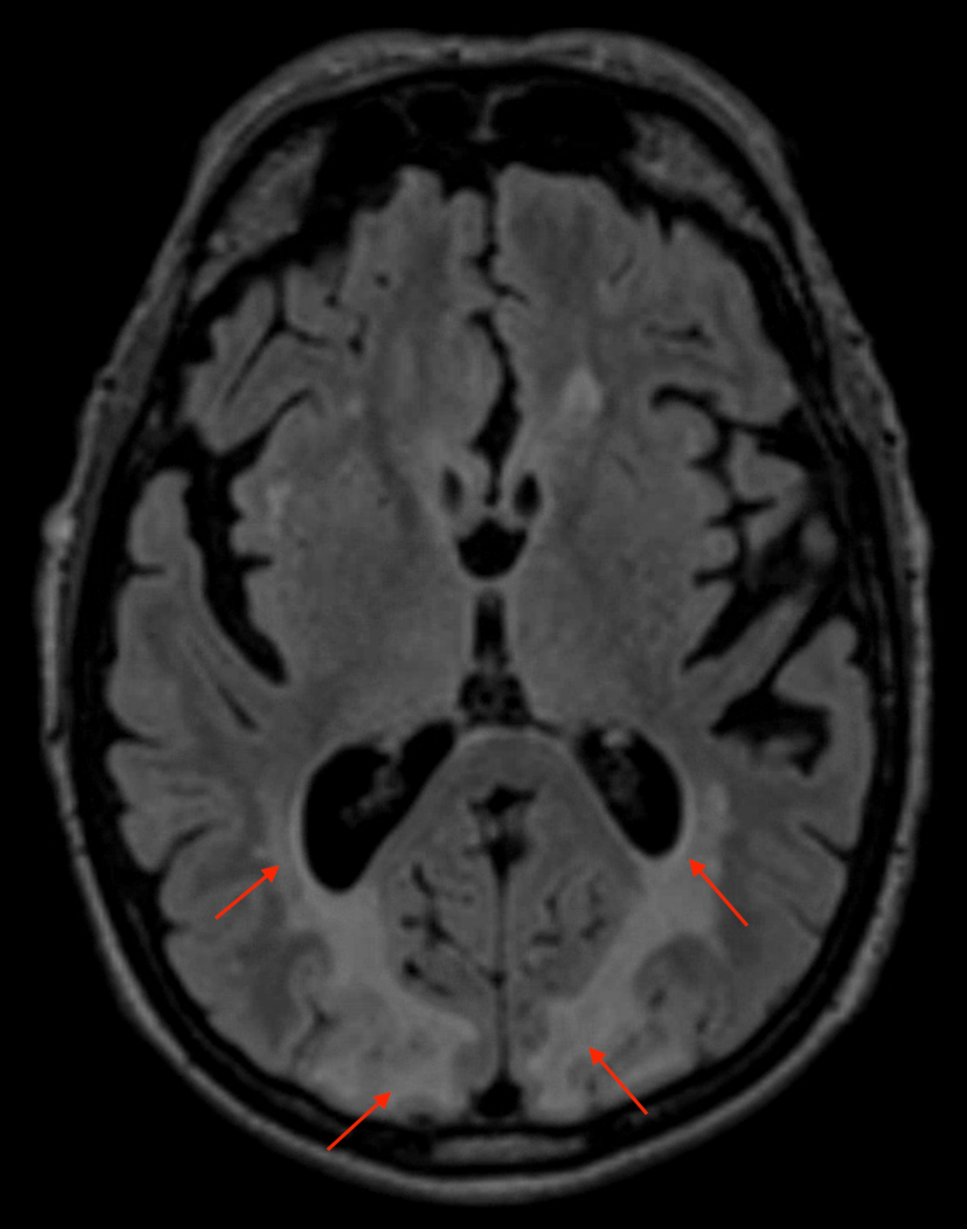
Fluid-attenuated inversion recovery (FLAIR) sequence MRI brain shows extensive oedema of occipital lobes

**Figure 3:**
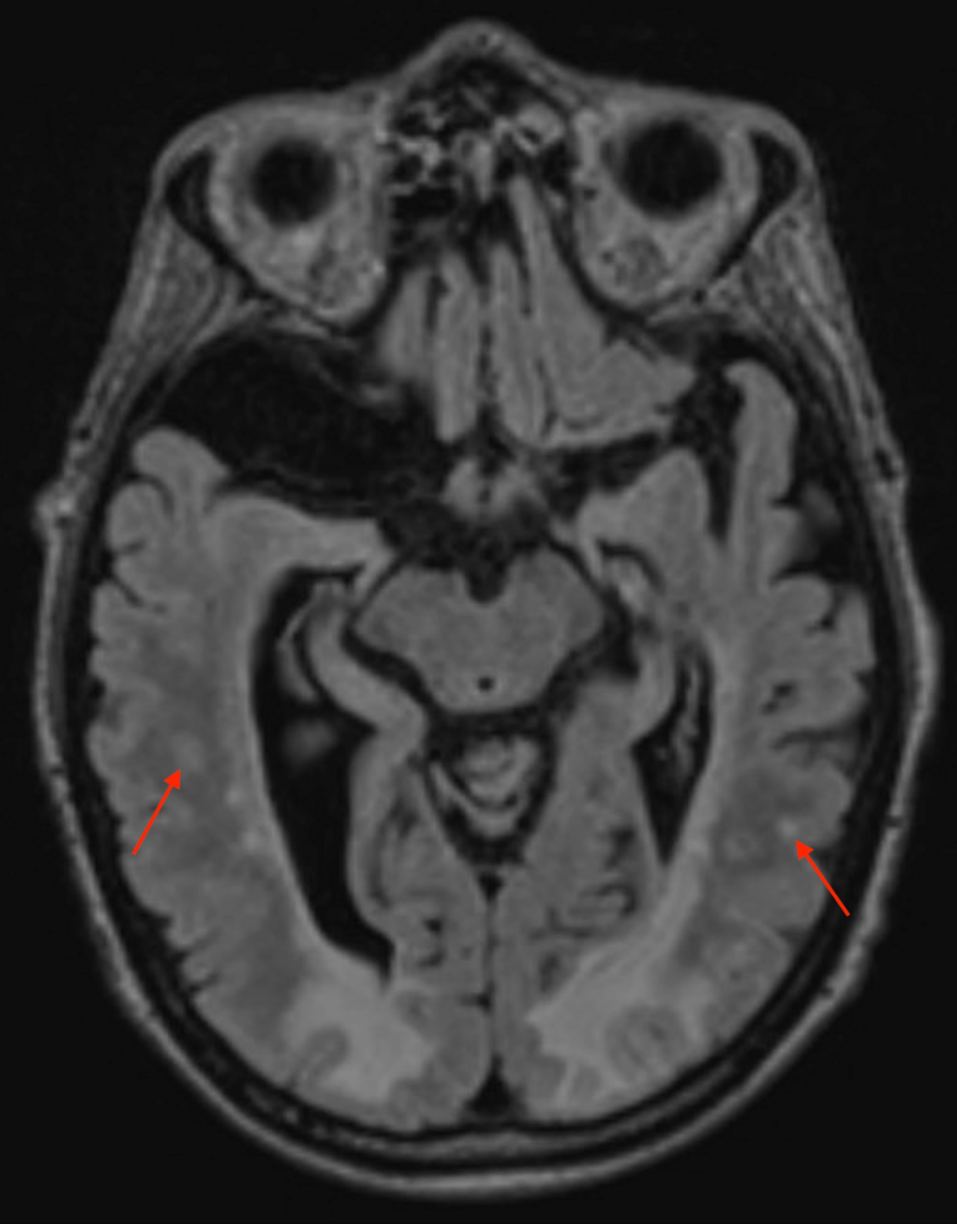
Fluid-attenuated inversion recovery (FLAIR) sequence MRI brain representing established small vessel ischaemic changes.

He subsequently received a CT of the head that revealed no acute intracranial infarction or haemorrhage; however, the occipital lobes showed diffuse and symmetrical low attenuation changes predominantly affecting the white matter (Figure 1). This imaging was able to provide a provisional diagnosis; however, it was later confirmed with MRI. MRI returned high signal on T2 and fluid-attenuated inversion recovery (FLAIR) sequences, which extensively and bilaterally affected the grey and white matter from the trigones of the lateral ventricles to the posterior occipital lobes (Figure 2). This represented widespread posteriorly located oedematous changes. Numerous hyperintensities were visible in the deep white matter representing established small vessel ischaemic changes (Figure 3).

Two days post-admission, the patient described progressively worsening visual symptoms and was found to have developed a left superior quadrantanopia whilst complaining of visual hallucinations. The patient described seeing hair on the bed and holes in the wall; however, fundoscopy did not reveal any intraocular pathology. His blood pressure was mildly elevated at 150 mmHg systolic on average, with periodic increases in excess of 180 mmHg systolic during his admission.

As the patient’s blood pressure was only mildly elevated despite withholding antihypertensives, they were not recommenced. However, despite further investigations and lack of evidence to support any precipitating cause for his development of PRES, mild hypertension was isolated as the sole potential cause of his symptoms. Subsequently, the patient received increasing titrations of amlodipine and additional ramipril with aim to reduce the blood pressure and alleviate his symptoms. 

Following the increased dose of antihypertensives, his symptoms started to resolve and following a short admission, in which he also received rehabilitation from allied health professionals, he was discharged. He was subsequently maintained on a higher dose regimen of antihypertensives for life to avoid future complications. Two weeks post-discharge, his symptoms had resolved and his blood pressure had stabilised at an average 119/68 mmHg.

## Discussion

Due to the vague presentation of this patient, most notably the visual symptoms and incoordination, a potential diagnosis of posterior circulation stroke was made. Whilst this may have been incorrect, it cannot be ruled out as a strong initial diagnosis for this patient due to the patient's past medical history of ischaemic heart disease and hypertension. It is also important to note that the presence of visual symptoms and an acute deterioration in cognition further strengthened the diagnosis of posterior circulation stroke, leading to treatment as per the stroke pathway. A major factor in the management of stroke patients is to maintain a slightly elevated blood pressure, which allows adequate perfusion of brain tissue and helps prevent further ischaemic damage [7]. This potentially may have led to some improvement of the patients symptoms; however, as this was a case of PRES, the patient's symptoms were in fact exacerbated. The pathophysiology of PRES demonstrates that the increase in blood pressure causes hyperperfusion of cerebral tissues, resulting in subsequent damage. This leads to a leakage of fluids causing vasogenic oedema. This oedema can be detected upon imaging, and it is difficult to ascertain whether this is a case of PRES without radiological assistance. Prompt diagnosis allows the correct management to be initiated. The distinction between PRES and stroke is vital as they both have distinct managements that can alter prognoses. Misdiagnosis and delays in treatment for either condition can be associated with increased morbidity and extended hospital stays, and most importantly may lead to the development of permanent sequelae [8].

Another diagnosis that was initially considered was an acute delirious episode. The patient had been diagnosed with mild vascular dementia and had numerous risk factors that are clinically associated with delirium. There was no identifiable cause for delirium as an infection screen revealed no growth in peripheral blood and urine cultures, with inflammatory markers within normal limits. There was no reported previous history of excessive alcohol consumption; however, liver function tests and ammonia levels to indicate hepatic encephalopathy or impairment were also within normal limits. In an absence of a firm diagnosis, it was important to rule out conditions that may cause further deterioration to ensure the safety of the patient. The previous diagnosis of vascular dementia coupled with mild hypertension may have acted in synchronisation as a potential precipitant for PRES. It is possible that in this group of patients, PRES can occur at lower blood pressure levels as they already demonstrate cerebral vessel damage. 

For the vast number of PRES cases, the cause is mostly acute hypertensive crisis, impaired kidney function or immunosuppression. Usually a blood pressure of around 180/110 mmHg is needed to establish an acute hypertensive crisis. Classically, a patient presenting would have severely raised blood pressure, which in turn lead to cerebral vascular damage and therefore cause symptoms that are described above. However, in this case, the patient maintained a systolic pressure of around 150 mmHg. There were only two brief episodes of the systolic pressure increasing to 180 mmHg. Despite the patient experiencing only a mildly raised blood pressure, it was concluded that this was most likely the cause for PRES in this patient. This hypothesis was strengthened by improvement of symptoms on initiation of antihypertensives.

Diagnosis, management and rehabilitation for PRES patients remain largely multidisciplinary [9]. Although initial admission to the acute stroke unit, medical advice regarding diagnosis and management was influenced by ophthalmologists, radiologists and neurologists. The altering visual signs in association with developing visual hallucinations were initially discussed with the ophthalmologists who were able to advise on the unlikeliness of intraocular pathology. Liaison with radiologists allowed for prompt imaging upon suspicion of PRES to give a definitive diagnosis. MRI scanning remains the definitive diagnostic imaging for PRES, and interpretation can differentiate an ischaemic cause from others, allowing the diagnosis of stroke to be excluded in this case. Upon establishing the diagnosis, discussion with neurologists advised an appropriate management plan through increasing antihypertensive medications. The involvement of multiple medical departments highlights the importance of the multidisciplinary approach in diagnosing this condition. Although PRES treatment can often be relatively simple to treat, arriving at the correct diagnosis often requires specialist input. Although most cases of PRES have a good prognosis, delays in management may lead to irreversible damage requiring prolonged rehabilitation and increased morbidity. It is also important to highlight the input from occupational and physical therapists in the rehabilitation of PRES patients to ensure they have reached their baseline prior to discharge. Discharging patients early with this condition can lead to further complications as the cognition can become severely impaired as demonstrated in this case. A combined holistic approach is necessary to manage this condition from initial presentation until discharge.

## Conclusions

This study presented the case of an 86-year-old gentleman with progressively worsening visual symptoms, decreased cognition and worsening cerebellar symptoms with an eventual diagnosis of PRES. In an absence of clear precipitating causes for PRES, it is important to consider mild hypertension in patients with a background of vascular dementia, as it may potentially increase the risk of developing PRES. Additionally, early involvement of the multidisciplinary team can lead to prompt diagnosis and treatment, thereby reducing significant morbidity and avoiding permanent complications.
